# Biodegradable Polymeric Membranes for Organic Solvent/Water Pervaporation Applications

**DOI:** 10.3390/membranes11120970

**Published:** 2021-12-09

**Authors:** Pao-Yueh Chang, Jane Wang, Si-Yu Li, Shing-Yi Suen

**Affiliations:** 1 Department of Chemical Engineering, National Chung Hsing University, Taichung 402, Taiwan; pychang124@gmail.com; 2 Department of Chemical Engineering, National Tsing Hua University, Hsinchu 300, Taiwan; janewang@mx.nthu.edu.tw; 3 Innovation and Development Center of Sustainable Agriculture, National Chung Hsing University, Taichung 402, Taiwan; 4 i-Center for Advanced Science and Technology, National Chung Hsing University, Taichung 402, Taiwan

**Keywords:** biodegradable membrane, pervaporation, poly(glycerol sebacate), poly(1,3-diamino-2-hydroxypropane-co-polyol sebacate)

## Abstract

Biodegradable polymers are a green alternative to apply as the base membrane materials in versatile processes. In this study, two dense membranes were made from biodegradable PGS (poly(glycerol sebacate)) and APS (poly(1,3-diamino-2-hydroxypropane-co-polyol sebacate)), respectively. The prepared membranes were characterized by FE-SEM, AFM, ATR-FTIR, TGA, DSC, water contact angle, and degree of swelling, in comparison with the PDMS (polydimethylpolysiloxane) membrane. In the pervaporation process for five organic solvent/water systems at 37 °C, both biodegradable membranes exhibited higher separation factors for ethanol/water and acetic acid/water separations, while the PDMS membrane attained better effectiveness in the other three systems. In particular, a positive relationship between the separation factor and the swelling ratio of organic solvent to water (DS_o_/DS_w_) was noticed. In spite of their biodegradability, the stability of both PGS and APS membranes was not deteriorated on ethanol/water pervaporation for one month. Furthermore, these two biodegradable membranes were applied in the pervaporation of simulated ABE (acetone-butanol-ethanol) fermentation solution, and the results were comparable with those reported in the literature.

## 1. Introduction

Membrane separation has been a persuasive technology utilized broadly in industrial separation processes. Amid various membrane applications, pervaporation is a popular membrane approach commonly employed as an alternative to conventional separation processes, such as distillation, extraction, adsorption, etc. The principle of pervaporation, combining permeation and evaporation, is the separation of liquid solvents by partial vaporization through a nonporous or porous membrane, whereafter the vapor permeating through the membrane is removed by vacuum or sweeping inert gas in the permeate side [1,2,3]. Its driving force is the chemical potential difference between membrane upstream and downstream. Membrane pervaporation can provide profitable benefits: simple process design, straightforward operation, easy maintenance, compact space, low energy consumption, high product quality, and low pollution [4,5,6,7], leading to widespread applications, such as solvent dehydration, azeotropic solvent purification, removal of volatile organic compounds (VOCs) from aqueous streams, separation of liquid hydrocarbons, dehydration to intensify esterification reaction, and so on [1,2,3,4,5,6,7,8,9,10,11,12,13,14,15,16,17,18].

Nonporous membranes or asymmetric membranes with dense active layers are preferentially utilized in pervaporation process. Molecular transport across the dense pervaporation membrane is usually governed by solution-diffusion mechanism [3,7,17,19]. In general, pervaporation is effective and economical for removing the minor component in the feed stream to minimize energy consumption and maximize separation efficiency. Most popular pervaporation membranes are polymeric such as PVA (polyvinyl alcohol), PAN (polyacrylonitrile), PI (polyimide), CS (chitosan), sodium alginate, PDMS (polydimethyl-siloxane), PTFPMS (poly((3,3,3-trifluoropropyl) methylsiloxane)), PTMSP (poly(1-(trimethylsilyl)-1-propyne)), PEBA (poly(ether block amide)), prototypical polymer of intrinsic microporosity (PIM-1), etc. [3,7,18,19,20,21,22,23,24,25,26,27,28,29]. Inorganic membranes, such as graphene, zeolite, metal organic frameworks (MOFs), and ceramic materials (e.g., titania, alumina, zirconia, silicalite, etc.) can also be used for pervaporation [3,8,30,31,32,33,34], but they are used less often due to the high production expense. Associating with both the advantages of polymeric matrix and inorganic filler, the so-called mixed matrix membranes (polymeric membranes incorporating inorganic fillers) have drawn an increasing attention in effectively improving the pervaporation performance. The tested inorganic filler substances include zeolite, alumina, silica, graphene oxide, MOF, porous 2D or 3D-shaped nanomaterials, and other ceramic nanoparticles [3,7,35,36,37,38,39,40,41,42,43].

Although good polymeric pervaporation membranes have a long-term stability and could be reused many times, the eventually exhausted membranes, if not degradable, will become the secondary pollutants because the combustion of these solid membrane wastes will emit plenty of gas contaminants or their disposal will produce environmentally polluted landfills. Since lots of polymers are resistant to degradation, causing a very critical issue in universal waste management, biodegradable polymers, thus, become the preferred candidate for the base material of membrane. Biodegradable substances can undergo deterioration and completely degrade when exposed to microorganisms in aerobic/anaerobic processes; such a degradation process will ultimately leave environmentally friendly byproducts. They are expected to become a strong competitor to conventional plastics. In this study, two kinds of dense membranes made from biodegradable poly(glycerol sebacate) (PGS) and poly(1,3-diamino-2-hydroxypropane-co-polyol sebacate) (APS) (easy to synthesize from glycerol and sebacic acid, inexpensive, nontoxic, and with good mechanical properties) [44,45] were tested in the pervaporation process to investigate their effectiveness on separating several individual organic solvents (ethanol, isopropanol, n-butanol, acetone, and acetic acid) from water. The characteristic properties and pervaporation performance of PGS and APS were systematically explored, and the results were compared to those of the PDMS membrane for a clear evaluation. Moreover, the stability of these two biodegradable polymeric membranes over a long-time period on organic solvent/water pervaporation was inspected in order to identify their reusability. A more practical application on the pervaporation of simulated ABE (acetone-butanol-ethanol) fermentation solution for PGS and APS membranes was also examined in this work.

## 2. Materials and Methods

### 2.1. Materials

Commercially available PDMS (Sylgard^®^ 184 silicone elastomer kit) was purchased from Dow Corning (Midland, MI, USA). All the solvents used in this study, including ethanol (99.5%), isopropanol (99.5%), n-butanol (99.5%), acetone (95%), and acetic acid (99.8%), were supplied by ECHO Chemical (Toufen City, Miaoli County, Taiwan) and Aencore (Box Hill, VIC, Australia). They were used as received.

### 2.2. Membrane Preparation

For the fabrication of dense PDMS membrane, the base reagent of PDMS kit was mixed with the curing agent in the ratio of 10:1 without any solvent. The mixture was stirred for 15 min, followed by ultrasonic vibration for 5 min, and then kept still for another 30 min to remove the air bubbles. The mixture was poured on a clean PET substrate evenly using a doctor blade to form a liquid film. The liquid film with the PET substrate was placed in an oven at 70 °C for 6 h to prepare a dense membrane. After curing, the membrane was carefully peeled off from PET.

PGS and APS were synthesized via the methods reported previously [44,45] in Dr. Jane Wang’s Lab of National Tsing Hua University, Taiwan, and fabricated into the form of flat sheet.

### 2.3. Membrane Characterization

The membrane thickness and surface morphology were observed by Field Emission-Scanning Electron Microscopy (FE-SEM, JSM-6700F, Jeol, Akishima, Tokyo, Japan), while the tapping-mode Atomic Force Microscope (AFM, Dimension Icon, Bruker, Billerica, MA, USA) with ScanAsyst was employed to evaluate the surface topography over a membrane area of 5 μm × 5 μm. The RMS (root mean square roughness) and Ra (arithmetic average roughness) parameters were analyzed with the software (Nanoscope v6.11, Bruker Optoc GmbH, Ettlingen, Germany). Moreover, the functional groups of the prepared membranes were detected using Attenuated Total Reflectance-Fourier Transform Infrared Spectroscopy (ATR-FTIR, Spectrum Two, Perkin Elmer, Waltham, MA, USA).

The thermal property of membrane was studied via a Thermogravimetric Analyzer (TGA, VersaTherm HS, Thermo Fisher Scientific, Waltham, MA, USA) under an inert atmosphere from 110 °C to 800 °C at a heating rate of 10 °C/min. A sample of approximately 13–15 mg was used for the analysis. Moreover, to figure out the glass transition temperature (Tg) of the polymeric membrane, the measurement with a sample of 3–5 mg was carried out through a Differential Scanning Calorimeter (DSC 6220, Seiko, Chiba-shi, Chiba, Japan) under the inert atmosphere from –80 °C to 270 °C at a scanning rate of 20 °C/min.

The water contact angle measurement was performed by using the instrument FTA-125 (First Ten Angstroms, Portsmouth, VA, USA) to determine the surface hydrophilicity and water wettability of membrane.

### 2.4. Solvent Swelling Experiment

A dry membrane sample was weighed first, and later immersed in a specific solvent at 37 °C for 8 h. The membrane sample was taken out and weighed immediately after carefully wiping the liquid remained on the surface. The degree of swelling (DS) of the membrane in the solvent was defined by the following equation:

(1) $$DS(\%)=\frac{W_{s}-W_{d}}{W_{d}}\times 100\%.$$

where W_s_ and W_d_ are the weight of the solvent-swollen membrane and that of dry membrane, respectively.

### 2.5. Pervaporation Experiment

**Figure 1:**
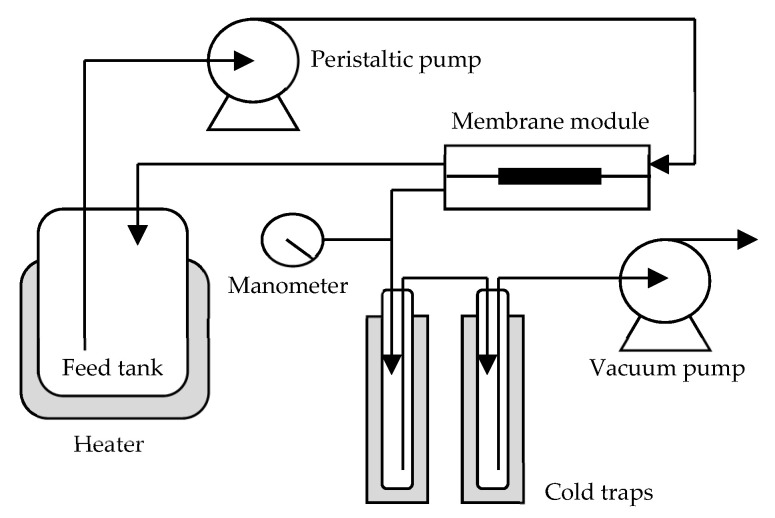
Schematic diagram of the pervaporation process in this study.

The schematic diagram of the pervaporation process in this study is illustrated in Figure 1. A total of 1 L of aqueous solution in the feed tank at 37 °C was circulated via a peristaltic pump at 300 mL/min across the membrane module (effective surface area: 19.63 cm^2^). The permeate was vacuumed (below 2 mmHg) and collected in cold traps (immersed in liquid nitrogen). After reaching steady state, the compositions of the aqueous solutions in the feed tank and the permeate were analyzed by GC equipped with a flame ionization detector (FID). The oven temperature was programmed from 100 °C to 150 °C at a rate of 20 °C/min. Both injector and detector temperatures were set at 225 °C.

The pervaporation performance is commonly examined in connection with the total permeation flux and separation factor. In this study, the total flux J was calculated by the following equation:

(2) $$J=\frac{W}{At},$$

where W is the total weight of the permeate (g), A is the effective membrane area (m^2^), and t is the duration time of the experiment (h). Considering that the permeation flux is usually affected by membrane thickness, a normalized flux J_N_ was expressed in terms of a certain membrane thickness L_N_ for normalization. The equation is as follows:

(3) $$J_{N}=\frac{JL}{L_{N}},$$

where L is the membrane thickness (μm) used in pervaporation process and L_N_ = 100 μm adopted in this study. The separation factor α was determined as:

(4) $$\alpha =\frac{y/(1-y)}{x/(1-x)},$$

where y and x represent the weight fractions of organic solvent in the permeate and the feed, respectively. Since a trade-off phenomenon between total permeation flux and separation factor typically occurs in pervaporation process, the overall membrane performance could, thus, be evaluated by combining these two important factors together, as the pervaporation separation index (PSI) [46]:

(5) $$PSI=J_{N}(\alpha -1).$$

## 3. Results and Discussion

### 3.1. Membrane Characterization

#### 3.1.1. Membrane Morphology

**Figure 2:**
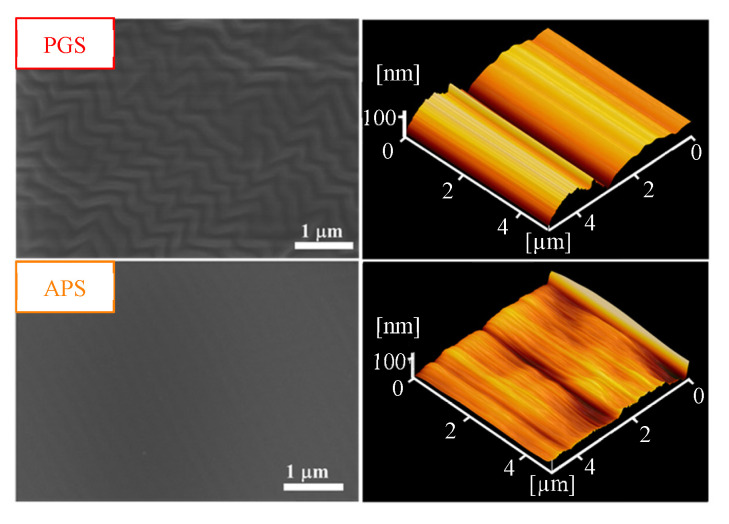
FE-SEM (×20S000) and AFM images of the two biodegradable membranes prepared in this study.

All the prepared membranes had a thickness of ca. 200 μm (degree of error <3%). The surface morphology and topography of each polymeric membrane were analyzed using FE-SEM and AFM, and the images of PGS and APS membranes are presented in Figure 2. Some patterns were revealed on the PGS membrane surface. Regarding the surface roughness parameters, the order was PGS (RMS = 17.65 nm, Ra = 13.67 nm) > APS (RMS = 12.50 nm, Ra = 8.71 nm) > PDMS (RMS = 7.98 nm, Ra = 4.92 nm). The PGS membrane had larger roughness values, which is attributable to its surface patterns. In comparison with the RMS data (60.5–370.2 nm) for several polymeric membranes (PDMS, CA (cellulose acetate), PES (polyethersulfone), PVDF (polyvinylidene fluoride), and sodium alginate/PVP (poly(vinyl pyrrolidone))) reported in the literature [47,48,49], all the three membranes prepared in this study showed much smaller surface roughness.

#### 3.1.2. ATR-FTIR Results

**Figure 3:**
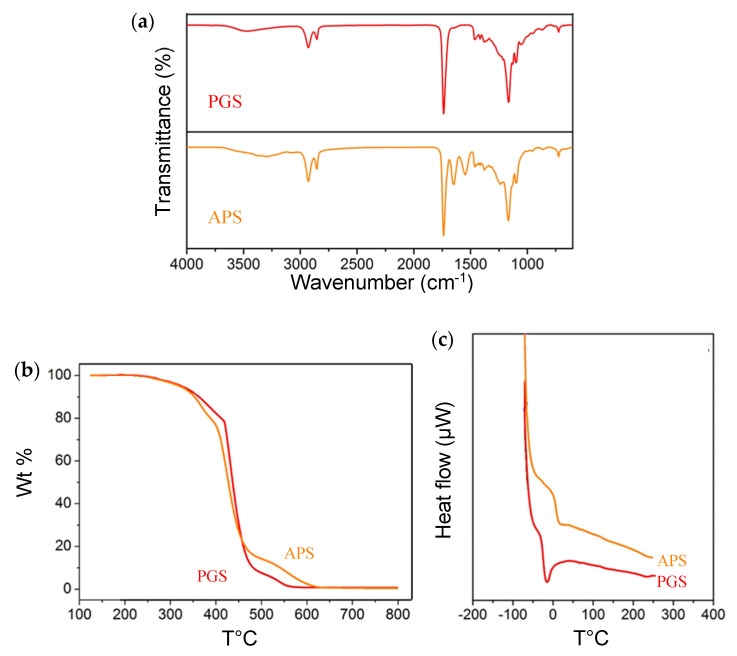
(**a**) ATR-FTIR spectra, (**b**) TGA plots, and (**c**) DSC patterns of the two biodegradable membranes prepared in this study.

The ATR-FTIR spectra of PGS and APS membranes are plotted in Figure 3a. These two biodegradable polyester membranes exhibited the characteristic CH and C=O peaks around 2960 cm^−1^ and 1740 cm^−1^ [50], while the APS membrane (amino alcohol-based poly(ester amide) [51]) displayed an additional NH peak at 1650 cm^−1^. Based on their general chemical structures [50,51], the ester and amide bonds of these two elastomer backbones were confirmed. As for the PDMS membrane (data not shown), its characteristic peaks were in good agreement with the literature data [27,47,52,53].

#### 3.1.3. Thermal Properties

Figure 3b shows the TGA plots of the two biodegradable polymeric membranes prepared in this study. Both PGS and APS membranes presented similar thermal stability: only a subtle weight loss from 110 °C to 300 °C, then a large weight reduction around 400–450 °C, and finally burned down to nil after 600 °C.

The DSC results of PGS and APS membranes are displayed in Figure 3c. The glass transition temperature (Tg) was estimated as –20 °C for PGS and 8 °C for APS. Both membranes had the Tg values lower than the operation temperature of pervaporation process (37 °C). This implies that these two polymeric materials were in a rubbery state during pervaporation, and the polymer chain flexibility could allow the vaporized solvent molecules to pass through the membrane more easily. Moreover, the polymeric membrane with a lower Tg (PGS) may have a higher chain mobility and hence a larger free volume. In comparison with the PDMS membrane (Tg ≈ −125 °C [29]), both biodegradable membranes had higher Tg values.

#### 3.1.4. Water Contact Angle Results

The water contact angle was measured for the as-prepared polymeric membranes. The results show that APS (85°) was more hydrophilic than PGS (94°), which should be attributed to the extra NH groups on the APS backbone. Moreover, the surface patterning and a little bit higher roughness of PGS membrane may have raised its hydrophobicity. On the other hand, these two biodegradable membranes had smaller water contact angle values than the PDMS membrane (104°).

#### 3.1.5. Swelling Behaviors

The solvent swelling effects at 37 °C for the two biodegradable membranes, along with the PDMS membrane, were investigated in this study. The results are shown in Table 1. All the three membranes exhibited much higher swelling degrees for organic solvents than for water, even in the case of APS membrane whose water contact angle (85°) was slightly less than 90°. Similar phenomenon was also revealed for pure chitosan membrane with a contact angle of 87° in the literature [54]. Since the solvent swelling degree could be correlated with the solvent solubility in the polymer [27], the results in Table 1 indicate that the PGS and APS membranes are preferable in hydrophobic pervaporation.

The DS values of APS for the three alcohols and water were slightly higher than those of PGS; on the contrary, the APS data for acetone and acetic acid were evidently smaller than the PGS results. These phenomena may be clarified in consideration of the hydrophobicity of organic solvent. Based on the Hansen solubility parameter (δ) [55,56], the hydrophobicity order is acetone (δ = 19.9) > acetic acid (δ = 21.4) > n-butanol (δ = 23.2) > isopropanol (δ = 23.6) > ethanol (δ = 26.5) (δ = 47.8 for water). For the more hydrophobic solvents, such as acetone and acetic acid, the APS membrane had far lower sorption degrees than PGS because it was more hydrophilic (water contact angle < 90°). However, its affinities with the three alcohols were reversed and became slightly stronger than PGS, which may be caused via more hydrogen bonds from the additional NH groups of APS backbone with alcohols.

Furthermore, all the DS data of PDMS membrane were greatly lower than the two biodegradable membranes. The low-degree swelling behaviors for water (<2%) and n-butanol (<30%) at 40 °C had also been displayed in the literature [27] using PDMS membranes with four different cross-linkers. Our data are consistent with their results. The cross-linking structure of PDMS should be the possible cause for its low sorption degrees. The high cross-linking density would restrict the polymer chain mobility and result in less free volume [27]. It would, thus, become more difficult for liquid solvent molecules to penetrate the highly cross-linked PDMS structure, leading to lower swelling degrees.

Consider that, when the feed mixture is loaded and touches the frontal membrane surface, both solvents in the mixture will compete with each other to sorb into the membrane. The ratio of their swelling degrees should be a more important index accounting for pervaporation performance than the individual values of swelling degree. Hence, the ratio of DS_organic solvent_/DS_water_ was evaluated, and the data are listed in Table 1. Comparing the two biodegradable membranes, APS revealed lower DS_o_/DS_w_ ratios than PGS in all the five organic solvent/water pairs. The APS data on acetone/water and acetic acid/water systems were noticeably smaller owing to its less hydrophobicity and poorer affinities with acetone and acetic acid, as mentioned previously. In addition, these two biodegradable membranes exhibited larger DS_o_/DS_w_ values than PDMS for ethanol/water and acetic acid/water systems but lower results in the other three pairs. It may be worthy to indicate that ethanol (4.3 Å) and acetic acid (4.4 Å) have smaller kinetic diameters than the other three organic solvents (acetone: 4.69 Å, n-butanol: 4.63–5.04 Å, isopropanol: 4.6–4.7 Å) [33,34]. The solvent swelling ratios did not only depend on the relative hydrophobicity of organic solvent to water (2.65–2.96 Å [33,34]) but also on their molecular sizes.

### 3.2. Pervaporation Performance

#### 3.2.1. Organic Solvent/Water Systems

The pervaporation process of organic solvent/water mixture was conducted at 37 °C using the membrane module with one piece of membrane disc. The results of the total permeation flux J and separation factor α are listed in Table 2. Moreover, the normalized permeation flux J_N_ in terms of 100 μm was also presented, for the later comparison with the literature data.

From the data in Table 2, the order of total permeation flux was PDMS > PGS > APS in most systems, except that PGS > PDMS > APS for isopropanol/water and PGS > APS > PDMS for acetic acid/water. The transport of solvent molecules across a dense membrane are principally governed by both their solubility and diffusivity with respect to the membrane [3,7,17,19]. Considering that PGS possessed greater hydrophobicity and higher chain mobility (presumed from the lower Tg value) than APS, the higher permeation flux was resulted for the PGS membrane in all the five organic solvent/water pervaporation cases. On the other hand, the PDMS membrane exhibited worse liquid solvent sorption degrees, but showed faster permeation in three pervaporation tests. It is more possible that the liquid solvent molecules were vaporized very soon after they dissolved into the upstream membrane surface. Thus, the vaporized solvent molecules would be able to pass through the cross-linked PDMS matrix more easily.

**Figure 4:**
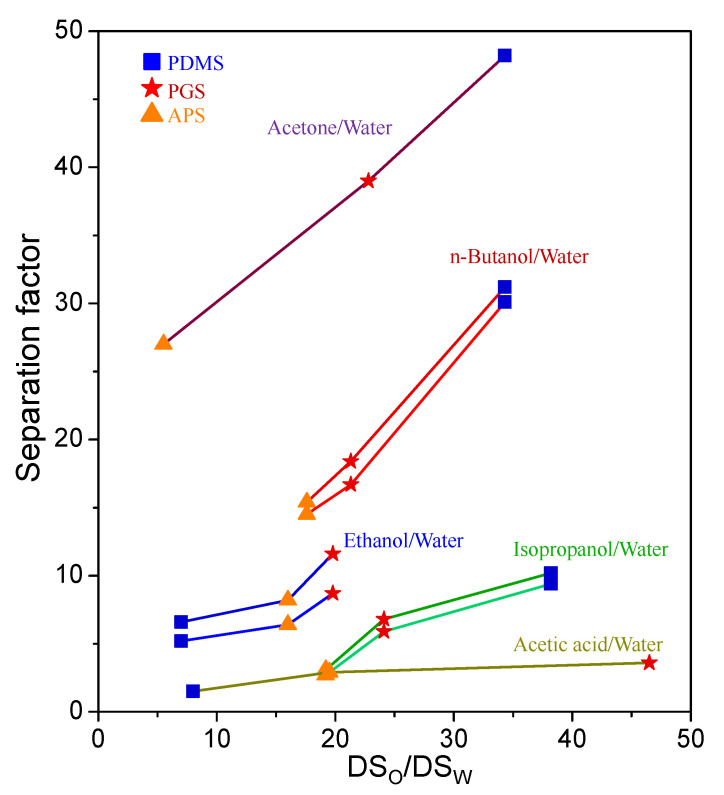
The relationship between the separation factor and the swelling ratio of organic solvent to water (DS_o_/DS_w_).

In Figure 4, the average value of separation factor in Table 2 was plotted versus the DS_o_/DS_w_ ratio in Table 1 to inspect their relationship and further understand the separation mechanism. A positive relation was attained: the greater the DS_o_/DS_w_, the higher the separation factor. The DS_o_/DS_w_ value may be interpreted as the ratio of organic solvent affinity with the polymeric membrane to water affinity with the polymeric membrane. An increase in DS_o_/DS_w_ corresponds to a higher membrane solubility for organic solvent in comparison with water, and henceforth, helps raising the concentration of organic solvent in the permeate. The separation factor is consequently improved. For all the five organic solvent/water systems, the PGS membrane presented better separation factor results than APS since it had higher DS_o_/DS_w_ ratios, which resulted from its larger hydrophobicity and good affinities with organic solvents. In all the cases, the PGS membrane exhibited the superior performance on both permeation flux and separation factor than APS. On the other hand, in comparison with the PDMS membrane, these two biodegradable membranes showed higher effectiveness for the separation of ethanol or acetic acid from water, both of which were the organic solvents with smaller kinetic diameters.

Furthermore, in the three alcohol/water systems, with the increasing feed alcohol wt%, the total permeation flux was enhanced, but the separation factor was decreased for all the membranes. The increase in total permeation flux could be explained by the much larger quantity of sorbed alcohol in membrane at a higher feed concentration and the promoted diffusion, as well as the less significant change in water flux [27,48]. This consequence led to a higher alcohol weight fraction in the permeate (y). However, when the alcohol weight fraction in the feed (x) was raised from 0.05 to 0.1 or 0.01 to 0.02, the enhancement in y/(1 − y) for the numerator of α (Equation (4)) was still smaller than the increase in x/(1 − x) for the denominator, resulting in a reduced separation factor (percentage decrease ≤ 25%).

As an overall index of separation efficiency, the PSI value of each membrane was calculated by combining the normalized total flux and separation factor together (Equation (5)). The PSI data are also displayed in Table 2. The order of PSI was almost identical to that of separation factor, except that the order became PGS > PDMS > APS for ethanol/water system. The separation factor is dominant in the whole separation effectiveness.

Table 3 summarizes the data of polymeric membranes applied to organic solvent/water pervaporations in the literature [27,47,57,58,59,60,61,62,63,64,65,66,67,68,69,70,71,72,73] for a comparison with the as-prepared polymeric membranes in this study. In most cases, our three membranes demonstrated excellent pervaporation efficiencies, especially superior to the literature results in the normalized flux and PSI value. For acetone/water and acetic acid/water systems, the separation factors of our membranes are even higher than those of the reported membranes. Only in the case of isopropanol/water pervaporation, the performances of our polymeric membranes are not as good as those of the PDMS-based membranes fabricated in the literature studies [60,64].

**Figure 5:**
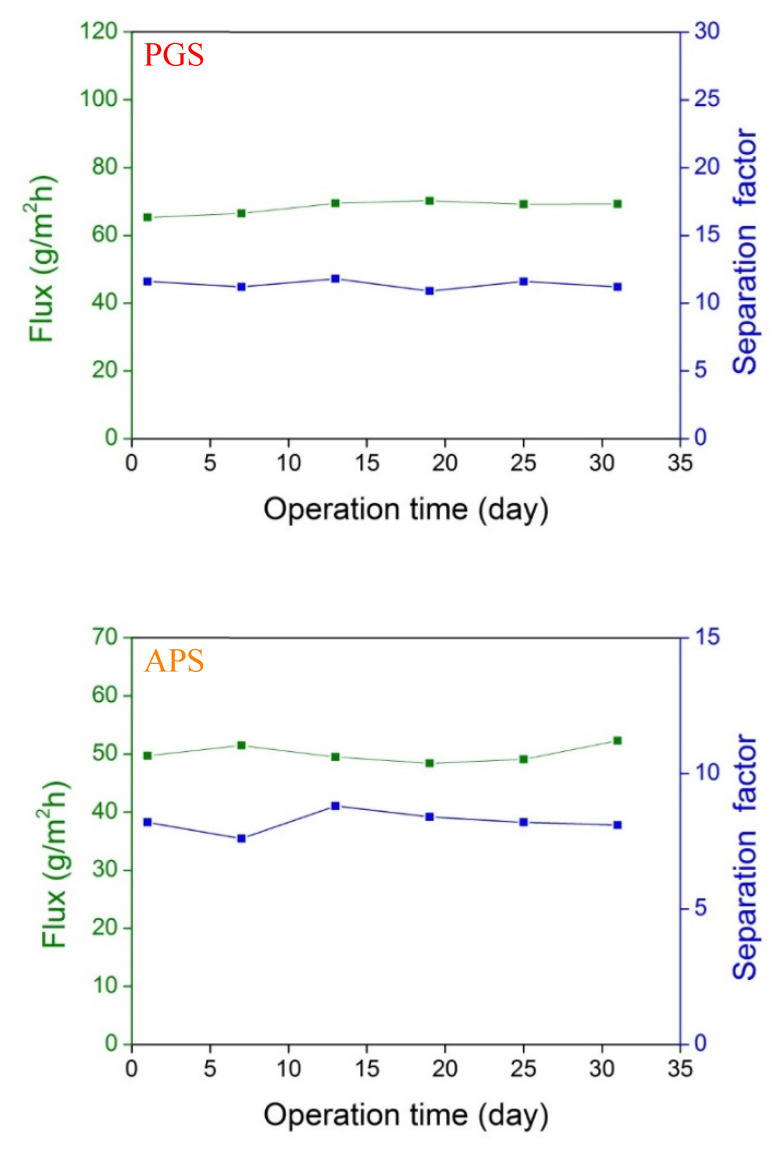
Ethanol (5 wt%)/water pervaporation performance at 37 °C using PGS and APS membranes for one month.

From the above analyses, the biodegradable PGS and APS membranes are very promising materials for achieving beneficial pervaporation performance, especially in the separation of ethanol/water and acetic acid/water mixtures. Although biodegradability is an attractive feature of these two membrane materials, it might cause a short lifetime and worsen the membrane reusability, eventually limiting their feasibility for practical applications. The PGS and APS membranes, synthesized mainly from glycerol and sebacic acid, have been known to degrade via hydrolysis and enzymatic degradation [74,75]. Their degradation rates via hydrolysis were reported to have a mass loss of ca. 17% after 60 days for PGS and around 13% after 20 weeks for APS [74]. To assess the long-term stability of these two biodegradable membranes on pervaporation process, their ethanol (5 wt%)/water pervaporation performance was monitored at 37 °C for 30 days. Both PGS and APS membranes reflected excellent stability during one month, as revealed by the nearly-unchanged flux and separation factor in Figure 5. The variations in data were less than 10%. Moreover, no impurities were detected on GC analysis, and none of the apparent membrane weight loss was found. Conclusively, these two biodegradable membranes could provide very good reusability for stable pervaporation performance, which are comparable to the literature results for PDMS-based membranes [27,47]. An additional benefit for these biodegradable membranes is that the membrane degradation would be easy to examine by either measuring the mass loss of dry membrane or detecting the presence of any degraded product in the permeate.

#### 3.2.2. Acetone–Butanol–Ethanol (ABE) System

To further explore the practical usage of PGS and APS membranes, a popular process of solvent recovery by pervaporation under the ABE fermentation [66] was simulated. The experiment was conducted at 37 °C in a working volume of 1 L with the aqueous feed solution containing acetone (0.7 g/L = 0.07 wt%), ethanol (0.4 g/L = 0.04 wt%), n-butanol (2.5 g/L = 0.25 wt%), and acetic acid (0.5 g/L = 0.05 wt%). The results are presented in Table 4, along with the data using PDMS/PAN/silicatite-1 mixed matrix membrane [66] and HTPB-based PUU membrane [70] for comparison. After normalization, both PGS and APS membranes exhibited higher total permeation flux J_N_ than both PDMS/PAN/silicatite-1 and HTPB-based PUU membranes. The selectivities towards ethanol and acetic acid for these two biodegradable membranes were better than those of the PDMS/PAN/silicatite-1 membrane, while their separation factors for acetone and n-butanol were superior to the HTPB-based PUU membrane. These phenomena were in good agreement with the previous results in Table 2 and Table 3: both PGS and APS membranes overwhelmed the PDMS membrane on ethanol/water and acetic acid/water selectivities; they also exhibited better separation achievements on acetone/water and n-butanol/water systems than the HTPB-based PUU membrane. The above optimistic results have further demonstrated the practicability of PGS and APS membranes on pervaporation applications.

## 4. Conclusions

In this study, two biodegradable polymeric membranes PGS and APS were successfully applied in the pervaporation of five organic solvent/water systems. In all the cases, the PGS membrane exhibited higher permeation flux and larger separation factor than APS. In comparison with the popular PDMS membrane, the two biodegradable membranes showed more impressive effectiveness for ethanol/water and acetic acid/water separations. Moreover, a positive relation between the DS_o_/DS_w_ ratio and separation factor was observed, which may be used as an indicator for the assessment of pervaporation performance. Although biodegradability is an attractive feature for both PGS and APS membranes, their reusability was not deteriorated via a long-term stability test on ethanol/water pervaporation for a period of one month. Furthermore, the pervaporation for the simulated ABE fermentation solution using the two biodegradable polymeric membranes resulted in greater normalized flux and comparable solvent recoveries with the PDMS-based mixed matrix membrane and the HTPB-based PUU membrane in the literature. These positive results expand the feasibility of PGS and APS membranes for practical pervaporation applications.

## Figures and Tables

**Table 1:** Solvent swelling data (37 °C) of the three polymeric membranes prepared in this study.

Membrane	DS (%)					
	Ethanol	Isopropanol	N-Butanol	Acetone	Acetic Acid	Water
PGS	85.2	103.6	91.8	98.0	199.8	4.3
APS	89.6	107.5	98.4	31.6	109.2	5.6
PDMS	4.2	22.9	20.6	20.6	4.8	0.6
**Membrane**	**DS_o_/DS_w_**					
	**Ethanol/Water**	**Isopropanol/Water**	**N-Butanol/Water**	**Acetone/Water**	**Acetic Acid/Water**	
PGS	19.8	24.1	21.3	22.8	46.5	
APS	16.0	19.2	17.6	5.6	19.5	
PDMS	7.0	38.2	34.3	34.3	8.0	

**Table 2:** Pervaporation performance (37 °C) of the three polymeric membranes (thickness of 200 μm) prepared in this study.

Feed Mixture	Organic Solvent wt% in Feed	Membrane	J (g/m^2^h)	J_N_ (g/m^2^h)	α	PSI (g/m^2^h)
Ethanol/water	5	PGS	65 ± 3	130	11.6 ± 1.1	1378
		APS	50 ± 3	100	8.2 ± 0.4	720
		PDMS	89 ± 2	178	6.5 ± 0.7	979
	10	PGS	77 ± 1	154	8.7 ± 0.8	1186
		APS	69 ± 3	138	6.4 ± 0.2	745
		PDMS	102 ± 1	204	5.2 ± 0.2	857
Isopropanol/water	5	PGS	75 ± 1	150	6.8 ± 0.1	870
		APS	22 ± 2	44	3.1 ± 0.2	92
		PDMS	64 ± 1	128	10.2 ± 0.7	1178
	10	PGS	82 ± 3	164	5.9 ± 0.2	804
		APS	35 ± 3	70	2.7 ± 0.2	119
		PDMS	69 ± 1	138	9.4 ± 0.6	1159
n-Butanol/water	1	PGS	53 ± 2	106	18.4 ± 1.0	1844
		APS	49 ± 1	98	15.4 ± 0.5	1411
		PDMS	66 ± 3	132	31.2 ± 0.8	3986
	2	PGS	66 ± 3	132	16.7 ± 0.2	2072
		APS	52 ± 2	104	14.5 ± 0.4	1404
		PDMS	72 ± 4	144	30.1 ± 0.2	4190
Acetone/water	0.5	PGS	51 ± 2	102	39.0 ± 0.7	3876
		APS	48 ± 2	96	27.0 ± 0.6	2496
		PDMS	63 ± 4	126	48.2 ± 0.5	5947
Acetic acid/water	10	PGS	118 ± 4	236	3.6 ± 0.5	614
		APS	96 ± 4	192	2.9 ± 0.3	365
		PDMS	82 ± 3	164	1.5 ± 0.2	82

**Table 3:** Literature review of the pervaporation performance of polymeric membranes.

Mixture	Membrane	Thickness (µm)	T (°C)	Organic Solvent wt% in Feed	J (g/m^2^h)	J_N_ (g/m^2^h)	α	PSI (g/m^2^h)	Ref.
Ethanol/ water	PDMS	100	30	8	25	25	10.8	245	[57]
	PDMS	34	30	10	179	61	1.8	49	[58]
	PDMS	9	37	6	~700	63	~8.3	460	[47]
	PSI (P_D_ 5000, 94% PDMS)	10	60	10	560	56	10.6	538	[59]
	PDMS-b-PPO	-	60	5	3816	-	8.5	-	[60]
	PTMSP-2	14	30	6	500	70	16.5	1085	[61]
	PTMSP-4	25	30	6	340	85	19.9	1607	[61]
	Pebax 2533	30	23	5	117.5	35	2.5	53	[62]
	PEO/CS (8 wt%)	20	20	8	900	180	4.4	612	[63]
Isopropanol/ water	PDMS	-	30	4	306	-	13	-	[64]
	PDMS-b-PPO	-	60	5	3650	-	13.5	-	[60]
n-Butanol/ water	PDMS/PVDF	10	30	1	160	16	43.1	674	[65]
	PPhS/PDMS/PVDF	10	30	1	261	26	46.8	1191	[65]
	PDMS-PhTMS/PVDF	12	40	1	704	84	41.5	3402	[27]
	PDMS/PAN/silicatite-1	7	37	1	708	50	30	1450	[66]
	Pebax 2533	100	23	5	65	65	8.2	468	[62]
	Pebax 2533	-	35	2.5	~300	-	~25	-	[67]
	Thin-film silicone	50	30	1	52.8	26.4	42	1082	[68]
	PolyHFANB-base a-BCP81	1.7	60	1	~3500	60	~22	1260	[69]
	HTPB-based PUU	140	35	1	~10.5	14.7	~9	118	[70]
Acetone/ water	Pebax 2533	30	23	5	140	42	3.3	97	[62]
	HTPB-based PUU	140	35	0.5	~6	8.4	~12.5	97	[70]
	PVC/PS-F2.0	40	30	5	42	17	11	170	[71]
Acetic acid/ water	PDMS	95	35	10	~57	54	~1.35	19	[72]
	PDMS-AMEO/PES	-	40	10	90	-	2.1	-	[73]

PSI: polydimethylsiloxane-imide; PPO: polyphenylene oxide; PEO: poly(ethylene oxide); PPhS: polyphenylsiloxane; PhTMS: phenyltrimethoxylsilane; a-BCP81: PolyHFANB-PolyBuNB = 300-130 (HFANB: hydroxyhexafluoroisopropyl; BuNB: butyl norbornene); HTPB: hydroxyterminated polybutadiene; PUU: polyurethaneurea; PVC/PS-F2.0: 9:1 weight ratio of PVC (polyvinyl chloride) and PS (polystyrene) with 2 wt% organo clay fillers; AMEO: aminopropyltrimethoxy; PES: polyethersulfone.

**Table 4:** Pervaporation results of simulated ABE system using PGS and APS membranes in comparison with the literature data.

Membrane	Thickness (µm)	T (°C)	Organic Solvent wt% in Feed	J (g/m^2^h)	J_N_ (g/m^2^h)	α	PSI (g/m^2^h)	Ref.
PGS	200	37	Acetone 0.07 Ethanol 0.04 n-Butanol 0.25 Acetic acid 0.05	29 ± 2	58	37.1 ± 0.5 13.7 ± 0.5 16.6 ± 0.6 2.5 ± 0.2	2094 737 905 87	This study
APS	200	37	Acetone 0.07 Ethanol 0.04 n-Butanol 0.25 Acetic acid 0.05	22 ± 3	44	33.5 ± 0.3 9.9 ± 1.0 14.5 ± 0.7 1.2 ± 0.6	1430 392 594 8.8	This study
PDMS/PAN/ silicatite-1	7	37	Acetone 0.067 Ethanol 0.043 n-Butanol 0.196 Acetic acid 0.026	491	34	41.4 9.8 31.6 -	1374 299 1040 -	[66]
HTPB-based PUU	140	40	Acetone 0.5 Ethanol 0.1 n-Butanol 1.1	9.7	13.6	15.3 - 13.7	194 - 173	[70]

## Data Availability

Not applicable.
